# A refined procedure for esophageal resection using a full minimally invasive approach

**DOI:** 10.1186/s13019-022-01765-2

**Published:** 2022-03-04

**Authors:** Simon K. Ashiku, Ashish R. Patel, Brandon H. Horton, Jeffrey Velotta, Sora Ely, Andrew L. Avins

**Affiliations:** 1 Department of Thoracic Surgery, Northern California Kaiser-Permanente, 3600 Broadway, Oakland, CA 94611 USA; 2 Division of Research, Northern California Kaiser-Permanente, Oakland, CA USA

**Keywords:** Esophagectomy, Esophageal cancer, Minimally invasive surgery

## Abstract

**Objective:**

Newer minimally invasive approaches to esophagectomy have brought substantial benefits to esophageal-cancer patients and continue to improve. We report here our experience with a streamlined procedure as part of a comprehensive perioperative-care program that provides additional advances in the continued evolution of this procedure.

**Methods:**

All patients with primary esophageal cancer referred for resection to the Oakland Medical Center of the Kaiser-Permanente Northern California health plan who underwent this approach between January 2013 and August 2018 were included. Operative and clinical outcome variables were extracted from the electronic medical record, operating-room files, and manual chart review.

**Results:**

142 patients underwent the new procedure and care program; 121 (85.2%) were men with mean age of 64.5 years. 127 (89.4%) were adenocarcinoma; 117 (82.4%) were clinical stage III or IVA. 115 (81.0%) required no jejunostomy. Median hospital length-of-stay was 3 days and 8 (5.6%) patients required admission to the intensive care unit. Postoperative complications occurred in 22 (15.5%) patients within 30 days of the procedure. There were no inpatient deaths; one patient (0.7%) died within 30 days following discharge and three additional deaths (2.1%) occurred through 90 days of follow-up.

**Conclusions:**

This approach resulted in excellent clinical outcomes, including short hospital stays with limited need for the intensive care unit, few perioperative complications, and relatively few patients requiring feeding tubes on discharge. This comprehensive approach to esophagectomy is feasible and provides another clinically meaningful advance in the progress of minimally invasive esophagectomy. Further development and dissemination of this method is warranted.

**Supplementary Information:**

The online version contains supplementary material available at 10.1186/s13019-022-01765-2.

## Introduction

Esophageal cancer is the seventh most common cancer worldwide and the sixth leading cause of cancer-related death [1], reflecting its generally poor prognosis. In 2018 in the United States, there were over 17,000 incident cases and 15,000 deaths [2]. Overall 5-year survival is approximately 20% [3], and most patients will die within one year of diagnosis [4].

This situation creates a compelling need to minimize treatment-associated morbidity to provide esophageal cancer patients with the maximum quality of life for their often-limited expected survival. However, the primary treatment modality for esophageal cancer, surgical resection, has been associated with high morbidity and mortality. In a review of Medicare data between 1997 and 2003, esophagectomies (primarily open procedures) were associated with an inpatient mortality of 3.0% and a 30-day mortality of 14% [5].

In an effort to improve on the outcomes of esophagectomy, Cuschieri, et al. developed and were the first to report on a minimally invasive esophagectomy (MIE) approach in 1992 [6]. The technique was considered experimental until Luketich et al.’s landmark 2003 series of 222 MIE procedures, which were associated with an operative mortality of only 1.4% [7]. Since that time, the procedure has been widely adopted and has progressively evolved as thoracic surgeons continue to incorporate additional innovations into the technique.

Despite the advantages over conventional open resections, there remain important areas for continued improvement in the process and postoperative outcomes associated with MIE procedures. Recent data show that median hospital length of stay (LOS) remains at approximately one week for MIE [8–39]. Median intensive care unit (ICU) length of stay, when reported, is typically between 1 and 3 days for both open and MIE procedures [9, 12, 14, 16, 17, 20, 26, 27, 30–32, 36, 37, 39, 40]. Reported 30-day readmission rates are typically 9–15% [11, 18, 22, 32, 41, 42]. Although minimally invasive surgical approaches to esophagectomy have important advantages, reported complication rates remain in the range of 23–65% [8, 10, 12, 13, 15–17, 19, 21–29, 29, 32, 33, 35, 36, 38–40, 42–46]. Furthermore, transabdominal jejunostomy feeding tubes are generally placed for post-operative nutrition, potentially causing postoperative complications [47] and delaying return-to-baseline functional status; routine feeding tube placement is described in most protocols and in three large series, ≥ 84% of patients were discharged home with feeding tubes [20, 37, 40]. Hence, there continue to be opportunities for further development and enhancement of the MIE approach. In addition, substantial advances have been made in perioperative care, such as enhanced-recovery-after-surgery (ERAS) protocols, providing potential additional improvements in patients' overall surgical experience.

Building on the notable progress made by prior surgical innovators, we sought to improve preoperative, intraoperative, and postoperative aspects of MIE, with the goal of improving esophageal cancer patients’ morbidity, mortality, and return-to-baseline functional status. We report here our experience with a streamlined fully minimally invasive operative approach along with a comprehensive perioperative care program.

## Methods

### Study design

We conducted a retrospective review of consecutive cases of Kaiser Permanente Northern California (KPNC) patients who underwent this streamlined MIE procedure and perioperative-care program (described below) and detailed in the accompanying document (Additional file 1). KPNC is an integrated health plan serving 4.4 million members through a network of 21 medical centers in Northern California. The demographics of KPNC approximate the underlying demographics of the geographic region [48]. All study procedures were approved by the KPNC Institutional Review Board.

### Subjects

Eligible patients were KPNC members referred for esophagectomy who underwent this MIE procedure at the KPNC Oakland Medical Center by one of five board-certified attending thoracic surgeons between January 2013 and August 2018; additional eligibility criteria included a surgical indication of primary esophageal cancer, a requirement that the procedure was elective, that the surgery consisted of the MIE only (i.e., not combined with another procedure during the same surgery), and that the patient had 90 days of postoperative follow-up at the time of data analysis (patients who died within 90 days of surgery were not excluded). All patients meeting these eligibility criteria were included and full ascertainment of all relevant patients was achieved by a search of the electronic medical record (EMR), operating-room records, and a list maintained by the first author. All patients who underwent the procedure in the specified interval were included in the final cohort, including those with extremely advanced disease for whom surgery was performed at the patient's request and were not expected to survive. Postoperative follow-up for surgical outcomes (including complications and mortality) was carried out for 90 days postoperatively on all patients; mortality follow-up was carried out to a maximum of 84.0 months (median: 29.0 months; survival curves were truncated when fewer than 10 patients remained at risk); data on mortality were obtained from the EMR and state and federal mortality databases.

### Description of perioperative and operative procedures

Perioperative care was guided by strict adherence to ERAS protocols, which included patient education with preoperative nutritionist consultation, mandatory perioperative medicine clinic consultation, carbohydrate loading with clear liquids 2-to-4 h preoperatively, avoidance of perioperative use of sedating medications, and use of nonopioid multimodal analgesia. Postoperatively, the urinary catheter and nasogastric tube were removed on the first day and a clear-liquid diet started and early ambulation instituted. Detailed descriptions of all aspects of the KPNC ERAS protocols are provided elsewhere [49, 50]. Most patients are discharged on postoperative day 3 (patients are eligible for discharge on day 3 if they have no leak on the esophagram, absence of tachycardia or fever, are able to sustain hydration and nutrition with over 2 L of fluid orally per day, their pain is adequately treated with oral medication, and they appear clinically well). Patients call or text the surgeon daily for 10 days after discharge after which they are seen in clinic; physician assistants assist in monitoring discharged patients.

The procedure described in this paper is classified as “IVL-LV” in the taxonomy of a recent consensus statement [51]. Briefly, the MIE in this study was performed in the following manner: The abdominal portion was conducted through a 15 mm, 10 mm, three mm ports and a 5 Nathanson liver retractor. The camera was operated by a surgical trainee. Both surgeon and assist employed two instruments. The hiatus was mobilized and Penrose drain placed, the greater curvature mobilized, the left gastric divided with a stapler and the stomach partially tubularized with three thick tissue stapler loads. Jejunostomy tube placement, the pyloroplasty, and the Kocherizaton of the duodenum were not done. To avoid trauma to the gastric conduit it was never grasped by instruments. The thoracic portion was conducted through four 10 mm ports and a 40–50 mm utility port. The pleura was incised caudal to cephalad and mobilized off the spine. The Penrose was retrieved at the hiatus and the esophagus and periesophageal tissue mobilized. A longitudinal incision was made in the upper esophagus to allow insertion of the anvil, the esophagus transected, and the anvil secured with two endoloop sutures. A gastrostomy was made on the lessor curvature and the stapler inserted. An anastomosis was created as proximal as possible and the tubularization complete leaving a 2 cm bridge. The pleura was closed with interrupted 2.0 silk sutures cranial to caudal.

### Variables

Patient-related data included demographics and clinical comorbidities (i.e., specific comorbidities and the Charlson-Quan Comorbidity Index summary score [52]), American Society of Anesthesiologists (ASA) preoperative risk score (range 1–5) [53], Eastern Cooperative Oncology Group (ECOG) performance-status score (range 0–5) [54], and neoadjuvant therapy received. Tumor-related variables included cell type, primary esophageal site, clinical and pathologic stage (consistent with the American Joint Committee on Cancer (AJCC)/Union for International Cancer Control (UICC) staging system, 8th edition, 2017 [55]) and regional lymph-node status. Variables related to the surgical procedure included both full operating-room time and “skin-to-skin” time (time between first incision and final closure), estimated blood loss, jejunostomy status, pleural-closure status and whether an intra-pyloric botulinum injection was used. Outcome variables included LOS for the overall hospitalization and for the ICU specifically as well as 30-day incidence of hospital readmission, emergency-department visits, whether the patient was discharged with a feeding tube in place, reoperation, and all-cause and cancer-specific mortality. Perioperative complications and post-discharge complications were all reviewed by the first author (SA) and categorized by the U.S. National Cancer Institute Common Terminology Criteria for Adverse Events (CTCAE) system, v5.0 [56].

### Data sources

Data for this study were obtained by extraction of relevant variables from the EMR database supplemented by manual chart review for those variables not available in discrete electronic form. Some cancer-related variables were obtained from a KPNC Cancer Registry maintained by the KPNC Division of Research for research purposes and for reporting to the Surveillance, Epidemiology, and End Results (SEER) program of the U.S. National Cancer Institute [57]. Data domains extracted from the EMR included demographics, clinical comorbidities, overall hospital ICU length of stay, readmission rates, and mortality. Variables requiring manual chart review included clinical and pathologic staging, lymph-node involvement, specific procedure-related variables, operative times from anesthesia records, and procedure-related complications. Manual chart data extraction was conducted by SA, AA, and BH.

### Data analysis

Continuous variables were summarized as means with Wald 95% confidence intervals and/or medians with the associated interquartile ranges (IQRs). Categorical variables were summarized with percentages and 95% confidence intervals (95% CI) were calculated with the exact Clopper-Pearson method [58]. Survival data for both total and cancer-specific mortality were summarized with Kaplan–Meier failure-time plots [59]. Comparisons of surgeries between the first and second halves of the cohort were conducted with nonparametric Wilcoxon rank-sum tests for continuous variables and Fisher's exact tests for categorical variables.

## Results

### Patients

One hundred fifty-two KPNC patients underwent this MIE procedure-and-care program between January 2013 and August 2018; of these, 10 patients were excluded for the following reasons: seven cases were complex procedures in which the MIE was combined with a second procedure during the same surgery (e.g., adrenalectomy, colon transposition, or lung lobectomy), two were unplanned salvage procedures, and one case was performed for recurrent esophageal cancer. The final analytic cohort was comprised of the remaining 142 patients (Table 1). No patient had a planned elective esophagectomy at this medical facility by any other procedure (e.g., open esophagectomy). All patients underwent the procedure for esophageal cancer, nearly all of whom had disease in the lower third of the esophagus or at the gastroesophageal junction (90.8%) and had adenocarcinoma histology (89.4%). Eighty-five cancers (59.9%) were clinical stage III and 32 (22.5%) were clinical stage IVA. The mean age of all patients was 64.5 years and the majority (85.2%) were men. One hundred twenty-five (88.0%) received some form of neoadjuvant therapy.

**Table 1:** Demographic and clinical characteristics of patients undergoing minimally invasive esophagectomy (N = 142)

Characteristic	
*Demographics*	
Age at time of surgery [years]	
Mean (SD)	64.5 (9.6)
Median (IQR)	66.5 (59–70)
Male gender [*N* (%)]	121 (85.2%)
BMI [kg/m^2^]	
Mean (SD)	27.4 (5.4)
Race [*N* (%)]	
African-American	7 (4.9%)
Asian	12 (8.5%)
Native American	1 (0.7%)
White	122 (85.9%)
Hispanic [*N (%)*]	10 (7.0%)
*Clinical characteristics*	
Primary site [*N* (%)]	
Mid-thoracic esophagus	13 (9.2%)
Lower thoracic esophagus	76 (53.5%)
Gastroesophageal junction	53 (37.3%)
Histology [*N* (%)]	
Adenocarcinoma	127 (89.4%)
Squamous cell carcinoma	11 (7.8%)
Other	4 (2.8%)
Clinical stage [*N* (%)]	
Stage 0	2 (1.4%)
Stage I	10 (7.0%)
Stage IIa	4 (2.8%)
Stage IIb	9 (6.3%)
Stage III	85 (59.9%)
Stage IVA	32 (22.5%)
Stage IVB	0 (0%)
Pathologic stage [*N* (%)]	
Stage 0	28 (19.7%)
Stage I	22 (15.5%)
Stage IIa	7 (4.9%)
Stage IIb	17 (12.0%)
Stage III	39 (27.5%)
Stage IVA	28 (19.7%)
Stage IVB	1 (0.7%)
Regional lymph nodes involved (Clinical staging) [*N* (%)]	87 (61.3%)
Regional lymph nodes involved (Pathologic staging) [*N* (%)]	53 (37.3%)
ECOG performance status [*N* (%)]	
0	42 (29.6%)
1	48 (33.8%)
2	12 (8.5%)
Unknown	40 (28.2%)
Neoadjuvant therapy [*N* (%)]	
Radiation therapy only	1 (0.7%)
Chemotherapy only	15 (10.6%)
Chemoradiation	109 (76.8%)
None	17 (12.0%)
Preoperative ASA Category [*N* (%)]	
1	0 (0%)
2	50 (35.2%)
3	91 (64.1%)
4	1 (0.7%)
5	0 (0%)
*Clinical comorbidities* [*N* (%)]	
Hypertension	87 (61.3%)
Diabetes	47 (33.1%)
Coronary artery disease	43 (30.3%)
Heart failure	8 (5.6%)
Chronic renal insufficiency	26 (18.3%)
Chronic obstructive pulmonary disease	13 (9.2%)
Stroke/Transient ischemic attack	4 (2.8%)
Charlson-Quan Comorbidity Index	
Mean (SD)	6.0 (5.6–6.4)
Median (IQR)	7 (4–8)

*ASA* American Society of Anesthesiologists, *BMI* Body Mass Index, *ECOG* Eastern Cooperative Oncology Group, *IQR* Interquartile range, *SD* standard deviation

### Operative outcomes

Of the 142 procedures, 1 required conversion to an open laparotomy due to intraoperative technical difficulties. The median total patient operating room time was 290 min (IQR: 255 to 348 min; mean: 306.5 min) and the median “skin-to-skin” time was 237 min (IQR: 210 to 290 min; mean: 254.6 min) (Table 2). One hundred fifteen patients (81.0%, 95% CI: 73.6% to 87.0%) did not have a jejunostomy placed during or after surgery.

**Table 2:** Operative parameter characteristics (N = 142)

Characteristic	
Intraoperative time (min)	
Full operating room time	
Mean (SD)	306.5 (73.7)
Median (IQR)	290 (255–348)
“Skin-to-skin” time	
Mean (SD)	254.6 (65.5)
Median (IQR)	237 (210–290)
Estimated blood loss (cc)	
Mean (SD)	142.0 (143.9)
Median (IQR)	100 (50–200)
Resection margin	
R0	128 (90.1%)
R1	14 (9.9%)^a^
Circumferential	11 (7.8%)
Longitudinal	3 (2.1%)
Lymph nodes excised (median (IQR))	15 (11–21)
Positive lymph node pathology* (%)*	37.3%
Jejunostomy [*N* (%)]	
Preoperative	18 (12.7%)
Intraoperative	3 (2.1%)
Postoperative	6 (4.2%)
None	115 (81.0%)
Pylorus injection of botulinum toxin [*N* (%)]	37 (26.1%)
Pleura closed [*N* (%)]	99 (69.7%)

IQR: Interquartile range, R0: clean surgical margins; no evidence of residual tumor, R1: evidence of residual tumor at surgical margin, SD: Standard deviation

^a^Two of these patients were alive and well at least four years after surgery, suggesting these patients likely had a complete resection with negative margins

### Clinical outcomes

The median hospital LOS was 3 days (IQR: 2 to 4 days); the mean was 3.6 days (95% CI: 3.1 to 4.0 days). Only 8 patients (5.6%, 95% CI: 2.5% to 10.8%) required postoperative care in the ICU (all were unplanned) and, of these, only 1 patient required care for more than 4 days (Table 3).

**Table 3:** Patient clinical outcomes (N = 142)

Clinical outcomes	N (%)
Hospital length of stay [days]	
Mean (SD)	3.6 (3.0)
Median (IQR)	3 (2–4)
Intensive care length of stay [N (%)]	
No intensive care days	134 (94.4%)
1–2 days	4 (2.8%)
3–4 days	3 (2.1%)
≥ 5 days	1 (0.7%)
Readmission within 30 days [N (%)]	14 (9.9%)
Post-discharge emergency department visit within 30 days [N (%)]	36 (25.4%)
Return to operating room within 30 days [N (%)]	6 (4.2%)
Required post-operative balloon pyloroplasty [N (%)]	33 (23.2%)
Mortality [N (%)]	
In-hospital	0 (0%)
Within 30 days of discharge	1 (0.7%)
31–90 days after discharge	3 (2.1%)
Patients with ≥ 1 complication within 30 days of surgery [N (%)]	22 (15.5%)
Highest CTCAE complication grade	
Grade 1	0 (0%)
Grade 2	8 (5.6%)
Grade 3	8 (5.6%)
Grade 4	4 (2.8%)
Grade 5 (death)	2 (1.4%)
Specific Complications	
Atrial fibrillation	4 (2.8%)
Anastomotic leak	3 (2.1%)
Dehydration	1 (0.7%)
Empyema	2 (1.4%)
Gastric conduit necrosis	1 (0.7%)
Myocardial infarction	1 (0.7%)
Pleural effusion	1 (0.7%)
Pneumonia	9 (6.3%)
Pneumothorax	1 (0.7%)
Respiratory failure	1 (0.7%)
Urinary tract infection (cystitis)	1 (0.7%)

*CTCAE* common terminology criteria for adverse events, *IQR* interquartile range, *SD* standard deviation

Twenty-two patients (15.5%, 95% CI: 10.0% to 22.5%) suffered a total of 25 complications within 30 days following the surgical procedure (Table 3); 14 patients (9.9%) experienced a complication with a CTCAE severity score of ≥ 3. The most common complications were pneumonia, atrial fibrillation, and anastomotic leaks (the frequencies of all complications are detailed in Table 3). In addition to these complications, 21 patients (14.8%) had a postoperative anastomotic stricture (i.e., that required dilation any time during the full extended follow-up period).

**Fig. 1:**
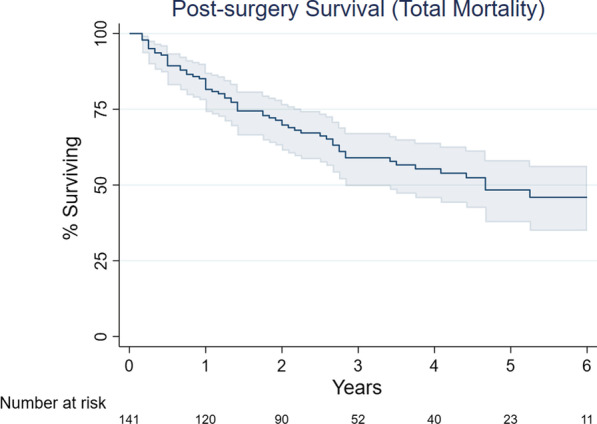
Kaplan–Meier overall survival curve for 142 patients undergoing minimally invasive esophagectomy described in this manuscript [point estimates (dark blue lines) with 95% confidence intervals (grey intervals)]; data truncated at 6 years when fewer than 10 patients had follow-up data after this point

**Fig. 2:**
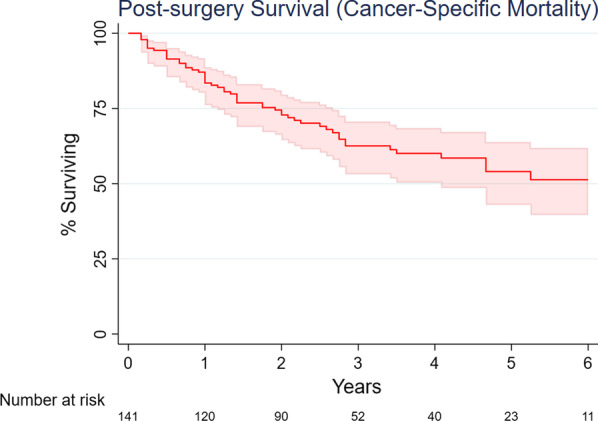
Kaplan–Meier curve for esophageal cancer-specific survival of 142 patients undergoing minimally invasive esophagectomy described in this manuscript [point estimates (dark red line) with 95% confidence intervals (pink intervals)]; data truncated at 6 years when fewer than 10 patients had follow-up data after this point

Within the 30 days after discharge, 14 patients (9.9%, 95% CI: 5.5% to 16.0%) were readmitted to the hospital. Thirty-six patients (25.4%, 95% CI: 18.4% to 33.3%) were evaluated in the emergency department, and 6 patients (4.2%, 95% CI: 1.6% to 9.0%) were taken back to the operating room (three for anastomotic leak, one for gastric conduit loss, one for empyema, and one for tracheostomy and jejunostomy to treat respiratory failure). No patient died intraoperatively or prior to discharge (0%, 95% CI: 0% to 2.6%); 1 patient (0.7%, 95% CI: 0.02% to 3.9%) died within 30 days post-procedure; 3 additional patients died between 31 and 90 days postoperatively (full 90-day mortality: 2.8%, 95% CI: 0.8% to 7.1%). The Kaplan–Meier product-limit estimate of the longer-term total mortality experience of this cohort is shown in Fig. 1 (total median survival was 4.6 years); cancer-specific survival is shown in Fig. 2.

Comparing surgeries performed in the earlier half of the cohort (prior to January 20, 2016) to the latter half, we found a reduction in the median hospital length of stay (4 days vs. 2 days, *p* < 0.001), median skin-to-skin procedure time (259 min vs. 229 min, *p* = 0.01), median number of lymph nodes excised (18 nodes vs. 14 nodes, *p* = 0.002), and risk of readmission within 30 days of discharge (11 (15.5%) vs. 3 (4.2%), *p* = 0.046).

## Discussion

Esophageal cancer is a deadly disease, with an overall 5-year survival of only 19% [4] and esophagectomy remains the mainstay of treatment. Since the introduction of the minimally invasive approach to esophagectomy in 1992 [6], there have been important improvements in the technique and outcomes associated with the procedure, though many patients continue to be burdened by surgical morbidity, lifestyle restrictions (e.g., use of feeding tubes), and prolonged hospitalization. Accordingly, thoracic surgeons have continued to build on prior innovations to further improve on the promise of MIE [60].

In this paper, we describe the process and clinical outcomes associated with a streamlined surgical approach to MIE developed at our institution among 142 consecutive patients with esophageal cancer treated with this procedure. This surgical technique was built around a fully minimally invasive approach incorporating updated techniques used by our group and others, motivated by prior advances in the procedure. In addition, we were careful to adhere to the principles of ERAS [49] with careful patient preparation and close post-discharge outpatient follow-up, further optimizing both the surgical and perioperative experience. No patient was lost to follow-up for the assessment of 90-day postoperative outcomes.

In conjunction with well-coordinated preoperative and postoperative care, we found this approach was associated with excellent outcomes, substantially improving objective measures of surgical and perioperative performance as well as the patient experience. In particular, we found that this comprehensive perioperative and surgical approach was associated with relatively shorter operative times, decreased need for both intensive-care and overall inpatient hospitalization, reduced need for feeding tubes, and acceptable adverse-event, reoperation, and readmission rates.

For example, we observed a median inpatient LOS of 3 days in our series with no patient requiring a routine ICU admission and only 5.6% requiring a subsequent transfer to the ICU for management of complications. These results compare favorably with prior published MIE studies with reported hospital LOS's between 7 and 33 days [8–40]; most studies reported LOS's of at least 12 days, with the largest case series reporting LOS’s of 8 to 15 days [18, 20, 22, 32, 35]. The great majority of studies that provided data on intensive care unit LOS's reported a median of at least one day [9, 12, 14, 16, 17, 20, 26, 27, 30–32, 36, 37, 39, 40, 61]. Furthermore, the elimination of a routine ICU admission and shorter overall LOS did not adversely affect rates of readmission or reoperation: our readmission rate was 12%, similar to previously reported rates of 9–18% [11, 18, 22, 32, 41, 42]. In addition, only 19% (95% CI: 12.9% to 26.4%) of our patients required placement of a jejunostomy tube, compared with reported rates of 84% [37], 95% [20], and 97% [40], among many other series that described routine jejunostomy-tube placement as part of the surgical protocol. We also found continued improvements comparing the earlier half of cohort with the latter half, suggesting that the learning curve for performing this procedure continued throughout the study period.

Comparing surgical complications across series is admittedly difficult since there is great variability in reporting standards. However, with respect to individual complications, we observed anastomotic leaks in 3 patients (2.1%, 95% CI: 0.4–6.0%) compared with others' reported rates that ranged from 0 to 21% [8, 10–12, 14, 16, 17, 19, 21, 23–40, 42–44, 61–63] with a median rate of 10% and a range from 5.5 to 21% among the five largest series that reported relevant rates [32, 35, 36, 42, 63]. In our series, pulmonary complications occurred in 14 patients (9.9%; 95% CI: 5.5% to 16.0%), including 9 cases (6.3%) of pneumonia and 2 cases (1.4%) of empyema; there were no cases of adult respiratory distress syndrome (ARDS) or chylothorax. These results compare favorably with rates from other centers with reported postoperative pneumonia rates ranging from 2 to 20% [10, 13–16, 19, 22, 25, 28, 29, 35–38, 40, 43, 44] with the median reported rate of 8%, and reported rates of empyema ranging from 0 to 4.1% [15, 20, 35, 37, 39, 42] with a median rate of 3.8%. Rates of ARDS reported in prior series range from 1 to 8% [8, 10, 16, 20, 23, 35, 40, 43] with a median of 3.3%; reported rates of chylothorax range from 1 to 11% [12, 14, 15, 17, 19, 23, 24, 26, 31, 32, 35, 37–40, 42, 44, 62] with the median reported rate of 3%. No patient died during the index hospitalization and 30-day mortality was 0.7% (95% CI: 0.02% to 3.9%) compared with reported rates in other series of 0–11% [9, 11, 12, 16, 18–20, 22–24, 32, 34–37, 40, 43, 45, 61, 63] (with most in the range of 2–4%).

The reasons for the favorable outcomes we observed were likely multifactorial. While the fundamental surgical principles remained unchanged for the esophagectomy itself, we streamlined several technical aspects, eliminating the need for the pyloroplasty, jejunostomy and Kocherization. Meticulous attention was made to avoid tissue trauma to the gastric conduit by utilizing a “no-grab” technique, implementing partial conduit tubularization, and avoiding a linear gastrotomy. Having two subspecialty thoracic surgeons working together further reduced time under anesthesia.

The decrease in hospital LOS reflects not only improved intraoperative techniques, but also intensive perioperative management. Saving the right pleura and reconstituting the mediastinal envelope eliminated the need for a feeding tube and allowed early removal of the nasogastric tube with resultant initiation of oral nutrition on the first postoperative day. Of particular note, only 19% of our patients required placement of a feeding jejunostomy tube compared to much higher rates reported in the literature. Employing a closed suction drain provided continued chest drainage, which could be continued as an outpatient to monitor for leaks. Postoperative pain management was simplified by administering long-acting intercostal nerve blocks thereby eliminating epidural catheters. Early alimentation and ambulation allowed the patient to recover earlier at home. There was daily telephone communication with a staff surgeon and on-demand access as necessary to monitor patients' progress and address any patient concerns. Return visits were mostly limited to drain removal. The combination of a streamlined procedure, strict adherence to ERAS protocols by a highly coordinated perioperative team, and close postoperative follow-up all likely contributed to the favorable patient outcome and experience.

Our case series has several strengths, including a consecutive closed cohort, complete 90-day follow-up, and detailed clinical and utilization EMR data. Our patient sample was typical of patients in other cohorts and trials in terms of age, gender, ASA classification, cancer stage, comorbidities, and use of neoadjuvant therapy. However, several limitations of this report should be noted. First, this study was based on a single-center, retrospective design. Additionally, this was a study of a streamlined surgical approach along with instituting a centralized multidisciplinary care method; therefore, it was not possible to determine the impact of each individual component on the improved outcomes. Although several surgeons performed the new procedure, suggesting the results are not limited to the practice of a single practitioner, generalizability will need to be validated in other practice settings. Finally, the retrospective data collection did not allow for assessment of standardized quality-of-life assessments.

## Conclusions

We found that an enhanced method of performing esophagectomy, employing a full minimally invasive approach, combined with a well-developed multidisciplinary perioperative-care program resulted in important improvements and excellent outcomes for the initial surgical treatment of esophageal cancer among the patients in our series. Further research should examine the generalizability of this approach and continue its development (ideally employing comparative clinical trials) to further improve the clinical experience and outlook for patients with esophageal cancer eligible for surgical resection.

## Supplementary Information

**Additional file 1: **Details of the operative and perioperative procedures for the minimally invasive esophagectomy procedure described in the text.

## Data Availability

The datasets used and analysed during the current study are available from the corresponding author on reasonable request.

## Abbreviations

AJCC: American Joint Committee on Cancer
ARDS: Adult respiratory distress syndrome
ASA: American Society of Anesthesiologists
CTCAE: Common Terminology Criteria for Adverse Events
ECOG: Eastern Cooperative Oncology Group
EMR: Electronic medical record
ERAS: Enhanced recovery after surgery
ICU: Intensive care unit
KPNC: Kaiser Permanente, Northern California
LOS: Length of stay
MIE: Minimally invasive esophagectomy
SEER: Surveillance, Epidemiology, and End Results
UICC: Union for International Cancer Control
