# Supplementary material for: A refined procedure for esophageal resection using a full minimally invasive approach
Source: J Cardiothorac Surg. 2022 Mar 4;17:29. doi: 10.1186/s13019-022-01765-2 (PMC8895824; doi:10.1186/s13019-022-01765-2)
Supplement: Supplementary file 1 — Additional file 1: Details of the operative and perioperative procedures for the minimally invasive esophagectomy procedure described in the text. [file 13019_2022_1765_MOESM1_ESM.docx]

**ADDITIONAL FILE 1**

**(Detailed description of operative and perioperative procedures)**

**Preoperative care**

On the day of tissue diagnosis, all patients are routed to a centralized team of oncologists who review the case and initiate a workup in conjunction with the local treating physicians. This involves ordering intravenous-contrast chest-and-abdominal computerized tomography (CT) and positron-emission-tomography (PET) scans and an endoscopic ultrasound study. All patient cases are then discussed and recommendations for care are finalized at a weekly, web-based, gastrointestinal (GI) malignancy multidisciplinary tumor board. The local oncologists and radiation oncologists are generally in attendance at the meeting and, when not, recommendations are communicated to them.

A programmatic, centrally based medical assistant helps coordinate appointments and assists the patient through the initial phase of the workup. Office appointments with the oncology and radiation oncology department are made at the nearest KPNC medical center. The thoracic surgeons are regionally based at a small number of KPNC facilities but have regularly scheduled office hours at the local hospitals near patients' homes. Nutritional status is enhanced by consultation with a local oncology nutritionist and, when indicated, an esophageal stent or jejunostomy tube is placed. For patients undergoing bi-modality or tri-modality therapy, chemotherapy and radiation therapy are administered locally. Patients are then restaged locally with a PET/CT scan and, when appropriate, an endoscopy.

Patients proceeding to surgery are referred to the Perioperative Medicine Service, a collaboration between anesthesiology and internal medicine physicians with specialty expertise in the identification and management of risk for perioperative patients. The Perioperative Medicine Service ensures that packages of care, such as the Enhanced Recovery After Surgery (ERAS) program are properly implemented, that each patient receives an individualized risk assessment to ensure that chronic medical conditions are optimized prior to surgery. This service also ensures that the decision to pursue surgical care is concordant with the patient’s goals.  All preoperative studies and tests are performed at the patient's local KPNC medical center.

The thoracic surgeon meets locally with the patient again for a final preoperative consultation encompassing preoperative education and assessment of the patient’s preparedness for surgery. All patients then receive an ERAS preoperation kit containing an incentive spirometer, chlorhexidine wipes, carbohydrate drink, and an information packet with important phone numbers and a “preparing for surgery” booklet.

**Intraoperative technique**

The surgical resection is a laparoscopic and thoracoscopic Ivor-Lewis esophagogastrectomy and is a modification of the technique described by Luketich, et al.^7^ This technique differs in several ways from established procedures. First, we eliminated the placement of a jejunostomy tube, creation of a pyloroplasty, and Kocherization of the duodenum. We added one additional 5 mm laparoscopic port in both the abdominal and thoracic portions to give the assistant surgeon a second working instrument. The operation of the surgical camera was assigned to a surgical resident, physician assistant, or medical student.

The process of tabularization of the gastric conduit was changed. Rather than fully tabularizing the conduit in the abdomen (which requires re-stitching the fundus of the gastric conduit to the remnant gastric cardia), we only partially tubularize the stomach in the abdomen, leaving the esophagus attached to the stomach. This enables pulling the gastric conduit into the chest without the additional step and trauma associated with suturing the specimen to the top of the gastric conduit. The final tubularization occurs in the chest after the anastomosis is created. In the technique described by Luketich et al., a linear incision is made in the gastric conduit (gastrotomy) through which a 28mm (or 25mm, if necessary) end-to-end anastomotic (EEA) stapler is introduced for the purpose of creating a gastric-to-esophageal anastomosis. Once the anastomosis is completed, the gastrotomy is sutured closed. To avoid this additional step and the trauma it causes to the gastric conduit, the gastrotomy is created on the lesser curvature of the stomach. The lesser curvature is then resected with the completion of tubularization, removing the traumatized gastrotomy portion of the stomach. Thus, creation of a gastrostomy in the gastric conduit, for introduction of the stapler, was also eliminated.

Several small modifications where made to reduce operative time. The diaphragm-retraction suture, used for better visualization of the hiatus, was eliminated since the extra 5 mm port allowed for diaphragmatic retraction. The placement securing the EEA stapler anvil into the esophagus was also modified for speed and simplicity. In the technique described Luketich, et al., the esophagus is completely transected at the site to be anastomosed and the anvil is inserted into the cut end of the esophagus where it is then sutured in place with two circumferential running sutures. The technique is complex and difficult and can traumatize the portion of the esophagus that is in the anastomosis. Rather than completely transecting the esophagus, we open it with a linear incision which enables simple introduction of the anvil. The esophagus is then transected, and the anvil secured with two endo-loop sutures ties. The traumatized distal portion of the cut esophagus used for the anvil insertion is then cleanly resected leaving healthy tissue around the anvil to be stapled. A 24F closed suction drain is added to allow patients to be discharged with a bulb suction drain for monitoring staple line leaks.

In a further departure from prior techniques, we added a step wherein we save, mobilize and reclose the right-sided mediastinal pleura over the anastomosed gastric conduit. The purpose of this maneuver is to contain the stomach within a mediastinal envelope to prevent it from becoming over distended with fluid or air. This allows us to remove the nasogastric tube (NGT) on the first postoperative day and to initiate oral feeding. In open and minimally invasive esophagectomies, the pleura is either resected or left open and NGTs are kept in place for up to one week to prevent gastric-conduit distention. The early oral feeds enabled us to eliminate the routine placement of jejunostomy tubes.

Finally, the entire operation was conducted with a “no-grab technique”. Prior to and after the creation of the gastric conduit, both in the abdomen and thorax, the stomach is never grabbed with instruments. Rather, it is gently pushed, pulled, lifted and rolled without grabbing it. This avoids unnecessary tissue trauma and capillary disruption of the gastric conduit which may have tenuous perfusion and relies on a capillary network to heal multiple staple lines. Liposomal bupivacaine mixed with 0.5% bupivacaine is used for multiple intercostal rib blocks. Local 0.5% bupivacaine solution is administered into the incisions.

**Postoperative care**

Patients are recovered in the post-anesthesia care unit and then transferred to a monitored surgical inpatient bed. Intravenous acetaminophen and hydromorphone are the initial pain medications. A diatrizoate (Gastrografin^®^) swallowing imaging study is obtained on the morning of the first postoperative day to assess gastric emptying and for identification of any possible anastomotic leak. The 28F chest tube is removed leaving only the 4F closed suction drain to bulb suction, and the urinary catheter and the NGT are removed. Patients are transitioned to an oral liquid hydrocodone/acetaminophen solution (15 cc every four hours) and an oral ibuprofen suspension, as needed. A clear liquid diet, including protein-fortified drinks, is started. Daily chest x-rays are obtained, but no routine blood tests are drawn. Essential home medications are restarted orally.

On the second postoperative day, the patient receives routine postoperative instructions from the nursing staff. The physician assistants educate the patients on their discharge diet and review the medical-center esophageal diet booklet. Patients are given a direct cellphone number and instructed to text or call the staff thoracic surgeon daily.

**Post-discharge care**

Daily phone calls or text reports are made to the staff thoracic surgeon directly from the patient. Patients report their fluid intake, heart rate, temperature and drain output. A protein-fortified clear liquid diet is maintained for 5 days, full liquid diet for 7 days, and then mechanical soft diet for 7 days. A regular diet then begins. Physician assistants call all patients the day after discharge and maintain communication as needed for general assistance. Patients return for a clinic visit with physician assistant staff (either at the regional center or locally) on postoperative day 10 for drain removal. The staff surgeon has a final telephone visit to assess recovery and to discuss the surgical pathology report. Patients are then returned to the care of their local oncologist.
